# The liver-alpha-cell axis after a mixed meal and during weight loss in type 2 diabetes

**DOI:** 10.1530/EC-21-0171

**Published:** 2021-08-11

**Authors:** Julia Otten, Andreas Stomby, Maria Waling, Elin Chorell, Mats Ryberg, Michael Svensson, Jens Juul Holst, Tommy Olsson

**Affiliations:** 1 Department of Public Health and Clinical Medicine, Umeå University, Umeå, Sweden; 2 Region Jönköping County, Jönköping, Sweden; 3 Department of Food, Nutrition and Culinary Science, Umeå University, Umeå, Sweden; 4 Department of Community Medicine and Rehabilitation, Section for Sports Medicine, Umeå University, Umeå Sweden; 5 NNF Center for Basic Metabolic Research and Department of Biomedical Sciences, University of Copenhagen, Copenhagen, Denmark

**Keywords:** amino acids, glucagon, hepatic insulin sensitivity, mixed meal, type 2 diabetes

## Abstract

**Objective:**

Glucagon and amino acids may be regulated in a feedback loop called the liver-alpha-cell axis with alanine or glutamine as suggested signal molecules. We assessed this concept in individuals with type 2 diabetes in the fasting state, after ingestion of a protein-rich meal, and during weight loss. Moreover, we investigated if postprandial glucagon secretion and hepatic insulin sensitivity were related.

**Methods:**

This is a secondary analysis of a 12-week weight-loss trial (Paleolithic diet ± exercise) in 29 individuals with type 2 diabetes. Before and after the intervention, plasma glucagon and amino acids were measured in the fasting state and during 180 min after a protein-rich mixed meal. Hepatic insulin sensitivity was measured using the hyperinsulinemic-euglycemic clamp with [6,6-^2^H_2_]glucose as a tracer.

**Results:**

The postprandial increase of plasma glucagon was associated with the postprandial increase of alanine and several other amino acids but not glutamine. In the fasted state and after the meal, glucagon levels were negatively correlated with hepatic insulin sensitivity (r_S_ = −0.51/r = −0.58, respectively; both *P* < 0.05). Improved hepatic insulin sensitivity with weight loss was correlated with decreased postprandial glucagon response (r = −0.78; *P* < 0.001).

**Conclusions:**

Several amino acids, notably alanine, but not glutamine could be key signals to the alpha cell to increase glucagon secretion. Amino acids may be part of a feedback mechanism as glucagon increases endogenous glucose production and ureagenesis in the liver. Moreover, postprandial glucagon secretion seems to be tightly related to hepatic insulin sensitivity.

## Introduction

Blood glucose levels are tightly regulated by insulin and glucagon. In type 2 diabetes, the ability of insulin to suppress endogenous glucose production is impaired, and increased fasting and postprandial glucagon levels stimulate endogenous glucose production even further (1, 2). Glucagon action is critical for correcting hypoglycemia but other potent stimulators of glucagon secretion are high amino acid levels, which may be an even more effective stimulus than low blood glucose levels (3). Thus, the concept of a liver-alpha-cell axis has recently been proposed to describe a feedback loop between amino acids and glucagon (4). Supporting this concept, inhibition of the glucagon receptor decreases amino acid catabolism in the liver, increases plasma amino acids levels, and causes alpha-cell proliferation and hypersecretion of glucagon (5).

It is a matter of debate which amino acids are responsible for signaling to alpha cells to increase glucagon secretion. Alanine and glutamine have been suggested to be key signal molecules within the liver-alpha-cell axis due to their high concentrations in the glucagon receptor knockout mouse and because glutamine may cause alpha cell proliferation (5, 6, 7). Alanine administered intravenously is a potent stimulator of glucagon secretion (8). In addition, a case report showed that plasma levels of glucagon and amino acids, particularly alanine, were increased in a female with a glucagon receptor mutation (9). Glutamine on the other hand is known to cause alpha cell proliferation in a cell culture model (7). In the perfused mouse pancreas, alanine, arginine, cysteine, glycine, lysine, and proline but not glutamine stimulated glucagon secretion (10).

Most plasma amino acids are elevated in type 2 diabetes (11). Moreover, higher fasting alanine levels have been associated with higher fasting glucose in cross-sectional studies (11, 12). In contrast, higher fasting glucose is associated with lower fasting glutamine levels (11). Thus, an assessment of the role of alanine and glutamine in the liver-alpha-cell axis in humans and particularly in type 2 diabetes is of obvious interest.

We aimed to evaluate the concept of the liver-alpha-cell axis in individuals with type 2 diabetes in the fasting state and after ingestion of a protein-rich meal. We investigated the association between the rise of the individual amino acids after the meal and the simultaneous rise in glucagon levels. We hypothesized that alanine, rather than glutamine, would be a predominant stimulator of glucagon secretion in patients with type 2 diabetes. It is well established that diet-induced weight loss improves hepatic insulin sensitivity in individuals with type 2 diabetes (13). However, the effect of weight loss on the liver-alpha-cell axis has not been investigated. We, therefore, evaluated the effect of a 12-week weight loss intervention (paleolithic diet ± exercise) on the liver-alpha-cell axis in individuals with type 2 diabetes. We hypothesized that postprandial glucagon secretion and hepatic insulin sensitivity are related and improved simultaneously.

## Methods

### Study design

Overweight patients with type 2 diabetes were randomized to either a Paleolithic diet alone or a Paleolithic diet combined with supervised exercise. At baseline and after 12 weeks of intervention, patients were examined with a solid mixed meal test, hyperinsulinemic-euglycemic clamping, and liver magnetic resonance spectroscopy. This paper represents a secondary analysis of hitherto unpublished data from a previously published study (14, 15). For the main analyses of this paper, the two intervention groups are combined and analyzed as one group, but we also report the effect of the different interventions on body composition, glucagon, and amino acids.

### Participants and randomization

Patients with type 2 diabetes (<10 years duration) and BMI of 25–40 kg/m^2^ were recruited by advertisements in local newspapers and posters at Umeå University Hospital (Umeå, Sweden) between 2012 and 2014. Study patients were 30–75 years of age, and women had to be postmenopausal. Exclusion criteria were treatment with glucose-lowering drugs other than metformin and severe illness. The primary outcome of the intervention study was a reduction in fat mass (14). Based on previous results from a similar study (16), we calculated that 13 individuals in each intervention group would be sufficient to detect a significant difference (*P* < 0.05) with 80% power. The results described in the present paper are secondary outcome measures. Patients were randomized to either the diet group or the diet and exercise group by a statistician not involved in the study, using biased coin minimization with an allocation ratio of 1:1. All examinations were conducted at Umeå University Hospital. The staff that performed the examinations and the blood sample analyses were blinded to the protocol and the dietary counseling. All patients gave written informed consent before study inclusion. The study protocol was in accordance with the Helsinki declaration and approved by the regional ethical committee of Umeå University. The study was registered in advance at ClinicalTrials.gov (NCT01513798).

### Diet intervention

A dietician instructed the patients how to follow a Paleolithic diet in five group sessions that were held separately for the two intervention groups. The Paleolithic diet was consumed without calorie restriction and was based on lean meat, fish, seafood, eggs, vegetables, fruits, berries, and nuts. It excluded dairy products, cereals, refined sugar, and salt. Dietary adherence was assessed with 4-day self-reported, weighed food records (14).

### Exercise intervention

All patients were recommended to increase their daily physical activity to at least 30 min of moderate-intensity activity. In addition, the diet and exercise group underwent a training protocol containing 1 h sessions of aerobic and resistance training three times weekly supervised by a professional trainer at the section of Sports Medicine at Umeå University.

### Solid mixed meal test

The study patients were instructed to refrain from physical exercise for 24 h before the test and to eat a standardized pre-specified meal at 19:00 h the day before and to remain fasting from 20:00 h. At 08:00 h the next day, patients visited the Clinical Research Department of Umeå University Hospital and were served a protein-rich Paleolithic-style breakfast. The meal included 70 g turkey, one boiled egg (ca 50 g), one banana (ca 105 g), half an apple with peel (ca 30 g), and cashew nuts without salt (25 g). The meal contained 419 kcal with 34%E carbohydrates, 41%E fat, and 25%E protein. Of the total protein content of the meal, 6% was alanine, and 16% was glutamine/glutamate. The percentage of all amino acids in the protein part of the mixed meal is shown in Supplementary Table 1 (see section on supplementary materials given at the end of this article). Blood samples were collected in pre-chilled tubes with 250 kallikrein inactivation units aprotinin before and 30, 60, 120, and 180 min after the meal. The samples were immediately centrifuged at 3000 ***g*** for 10 min at 4°C, and plasma was stored at −80°C until analysis.

### Plasma analyses and calculations

Plasma glucose was analyzed with Cobas GLUC3 (Roche Diagnostics; coefficient of variation 3%) and plasma insulin with the Bio-Plex Pro^TM^ Human Diabetes Assay (Bio-Rad Laboratories; coefficient of variation 7.5%) at the Department for Clinical Chemistry, Umeå University Hospital. In the same department, hemoglobin A1c was analyzed with TOSOH G8 (TOSOH Bioscience Inc, San Francisco, USA; coefficient of variation 3%) and blood lipids with Cobas Pro (Roche Diagnostics; coefficient of variation 3–4%). Radioimmunological determination of glucagon was performed as previously described using an assay directed against the COOH terminus, which reliably measures pancreatic glucagon (2, 17, 18). The analytical detection limit was 1 pmol/L. Normal range for fasting glucagon is 1–15 pmol/L. The intra assay coefficient of variation is <6% and the inter-assay variation < 15%. Amino acid analyses in plasma are described in the Supplementary Methods. The amount of plasma was limited during the mixed meal examination and not all analyses could be conducted in every participant. The area under the curve (AUC) was calculated using the trapezoidal rule and total AUC was used.

### Other examinations

Hepatic insulin sensitivity was measured using the hyperinsulinemic-euglycemic clamp technique combined with [6,6-^2^H_2_]glucose infusion and liver fat with proton magnetic resonance spectroscopy as described previously (15).

### Statistical analysis

**Figure 1:**
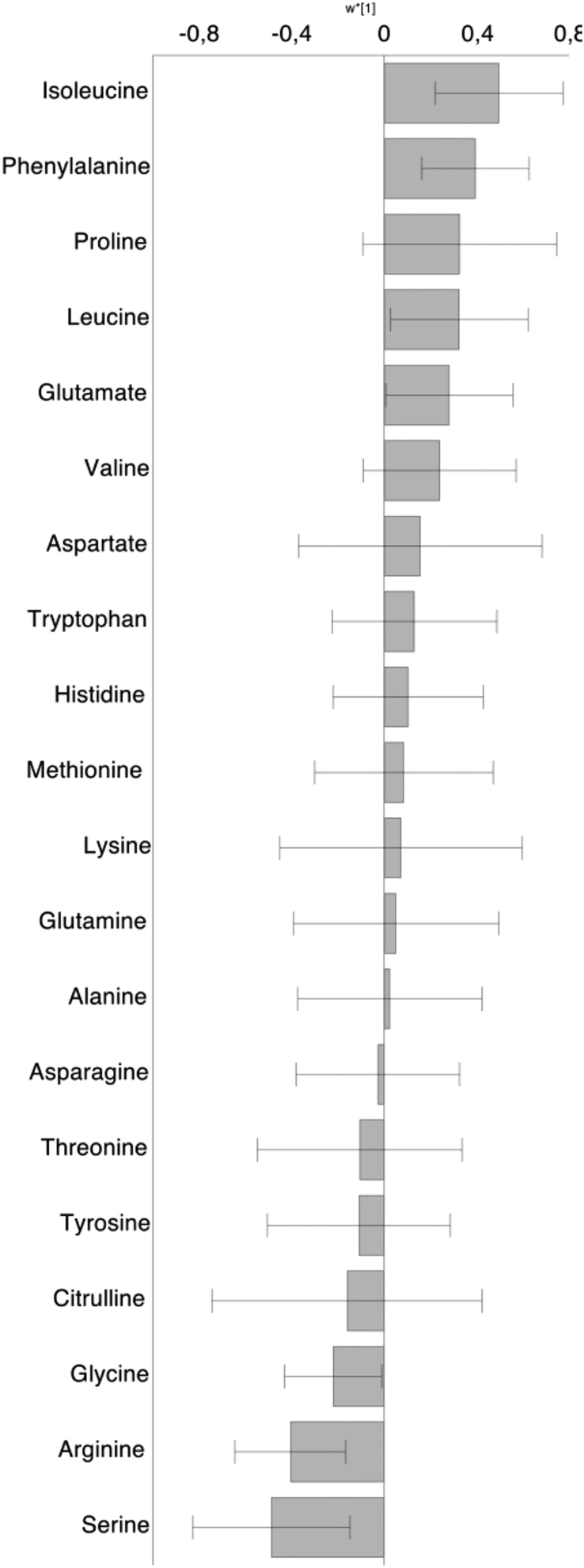
Association between fasting plasma glucagon levels and 20 different fasting plasma amino acids. Data are presented as multivariate regression coefficients (w*[1] according to OPLS) with 95% CIs. X-variables (=amino acids) with a 95% CI that does not include zero are significantly associated with the Y-variable (=glucagon). The amino acids are arranged depending on their strength of correlation to glucagon. Results are based on 27 fasting samples in altogether 13 different patients.

**Figure 2:**
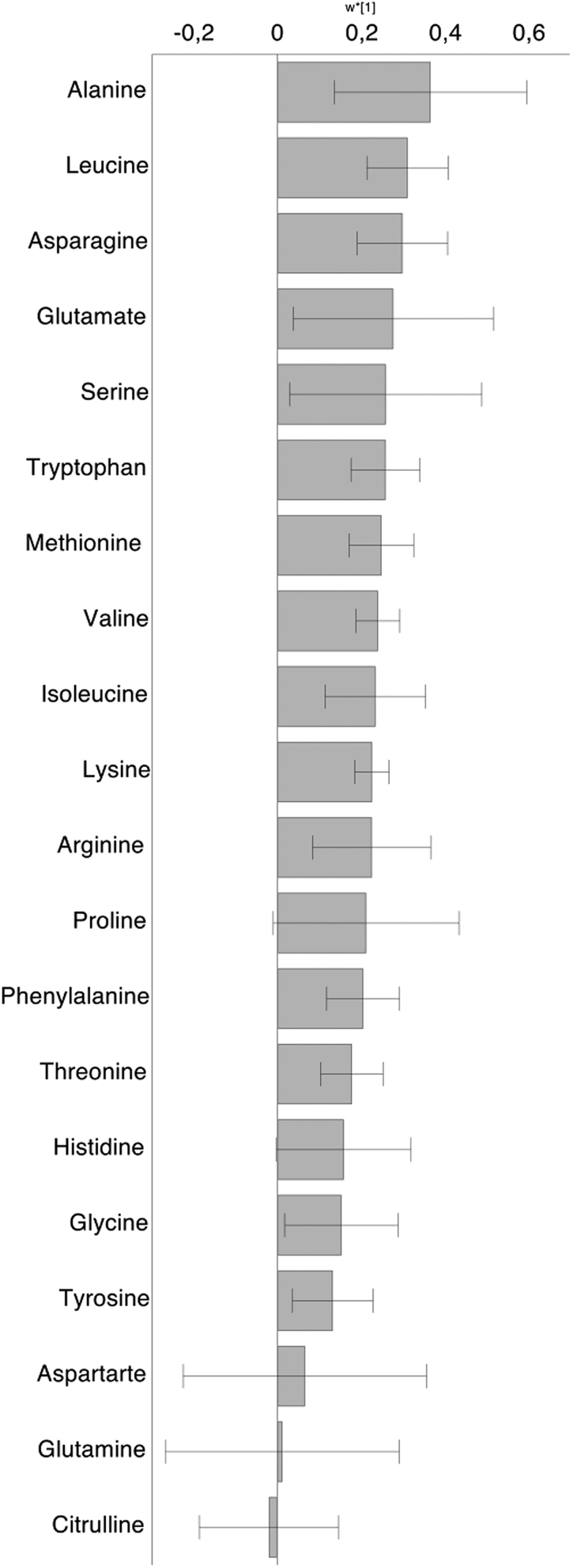
Association between the increase of postprandial plasma glucagon 0-60 min after a protein-rich mixed meal and the change of 20 different amino acids during the same time period. Data are presented as multivariate regression coefficients (w*[1] according to OPLS) with 95 % CIs. X-variables (=amino acids) with a 95% CI that does not include zero are significantly associated with the Y-variable (=glucagon). A positive value implies that the increase of this amino acid 0-60 min after the mixed meal is correlated to the increase of glucagon 0-60 min after the mixed meal. The amino acids are arranged depending on their strength of correlation to glucagon. Results are based on 25 mixed meal tests in altogether 14 study patients.

All variables were investigated with histograms to evaluate the pattern of distribution. None of the analyzed variables were normally distributed except for AUC glucagon at baseline and for the change during the intervention but not for the measurements at 12 weeks. Endogenous glucose production/insulin was normally distributed in the study population as a whole but not in the two separate intervention groups. We conducted dependent/independent *t*-tests for normally distributed variables. For the other variables, we used the Wilcoxon signed-rank test to analyze the change over time and the Mann-Whitney *U*-test to compare the two groups. Correlation analyses were performed using Pearson correlation (r) for the normally distributed variables and Spearman’s rho (r_S_) for the remaining variables. A two-sided *P* value < 0.05 was considered statistically significant. SPSS 25.0 for Mac was used for statistical analysis (IBM Corp). In one single multivariate analysis, all plasma amino acid levels were compared to plasma glucagon levels. Results for the fasting state are presented in Fig. 1 and the postprandial state in Fig. 2. For the postprandial analysis, the increase in all plasma amino acid levels between 0 and 60 min after the mixed meal was compared to the glucagon change during the same time. Here, individual measures before the mixed meal were subtracted from their measures 60 min after the mixed meal to minimize the influence of inter-individual variation. We applied a variant of orthogonal partial least squares (OPLS), for example, OPLS-effect projection for the analysis (19). The OPLS model was validated based on ANOVA of the cross-validated OPLS scores for significance testing (20). In each analysis, 25 mixed meal tests (performed before or after the 12-week intervention) of 14 different study patients were included. The OPLS model can cope with the multicollinearity of the data (11 patients contributed with two mixed meal tests to the analysis) and with the high number of variables (20 amino acids and glucagon) compared to the number of observations (25 mixed meal tests). Jack-knifing was used to calculate 95% CIs (21). X-variables (=amino acids) with a 95% CI that does not include zero are significantly associated with the Y-variable (=glucagon). All multivariate analyses were done using SIMCA 15.0 software (Sartorius, Umeå, Sweden).

## Results

Participants’ characteristics are given in Table 1. Despite the different inclusion criteria between men and women with regard to age, there was no significant difference in age between sexes. Women had a median age of 61 years (95% CI 59–66) and men of 59 years (95% CI 57–64). We evaluated the effects of the intervention on the liver-alpha-cell axis combining both intervention groups, as the effect on weight loss for the different analyses did not differ and because of insufficient power for doing separate analyses within the study groups. Effects of the different interventions on body composition, glucagon, and amino acids are presented at the end of the results section and in the supplement.

**Table 1:** Participants’ characteristics. Data are reported as the median (interquartile range).

	All
*n* (Male/female)	29 (19/10)
Age, years	61 (57–65)
BMI, kg/m^2^	31.4 (29.1–33.7)
Diabetes duration, years	3 (1–8)
HbA1c, mmol/mol (ref 31–47)	55 (48–58)
HbA1c, % (ref 5.0–7.7)	7.2 (6.5–7.5)
Systolic blood pressure, mmHg	133 (127–146)
Diastolic blood pressure, mmHg	84 (77–92)
Total cholesterol, mmol/L (ref 3.9–7.8)	4.3 (3.6–4.9)
LDL, mmol/L (ref 2.0–5.3)	2.2 (1.7–2.8)
Triglycerides, mmol/L (ref < 2.6)	1.9 (1.2–2.8)
Metformin therapy	20
Statin therapy	14

### The liver-alpha-cell axis in the fasted state

At baseline, fasting glucagon was correlated to total fasting amino acid levels (r_S_ = 0.67, *P* < 0.05). When analyzing all fasting amino acids individually with the multivariate model, isoleucine, leucine, phenylalanine, and glutamate were significantly and positively associated with fasting glucagon but that was not the case for fasting alanine and glutamine (Fig. 1). Fasting arginine and serine were negatively associated with fasting glucagon.

Fasting glucagon levels at baseline were associated with body weight (r_S_ = 0.56, *P* < 0.01) and with hepatic insulin sensitivity, measured as suppression of endogenous glucose production during the euglycemic-hyperinsulinemic clamp, normalized by insulin (r_S_ = −0.51, *P* < 0.05).

### The liver-alpha-cell axis after a protein-rich mixed meal

**Figure 3:**
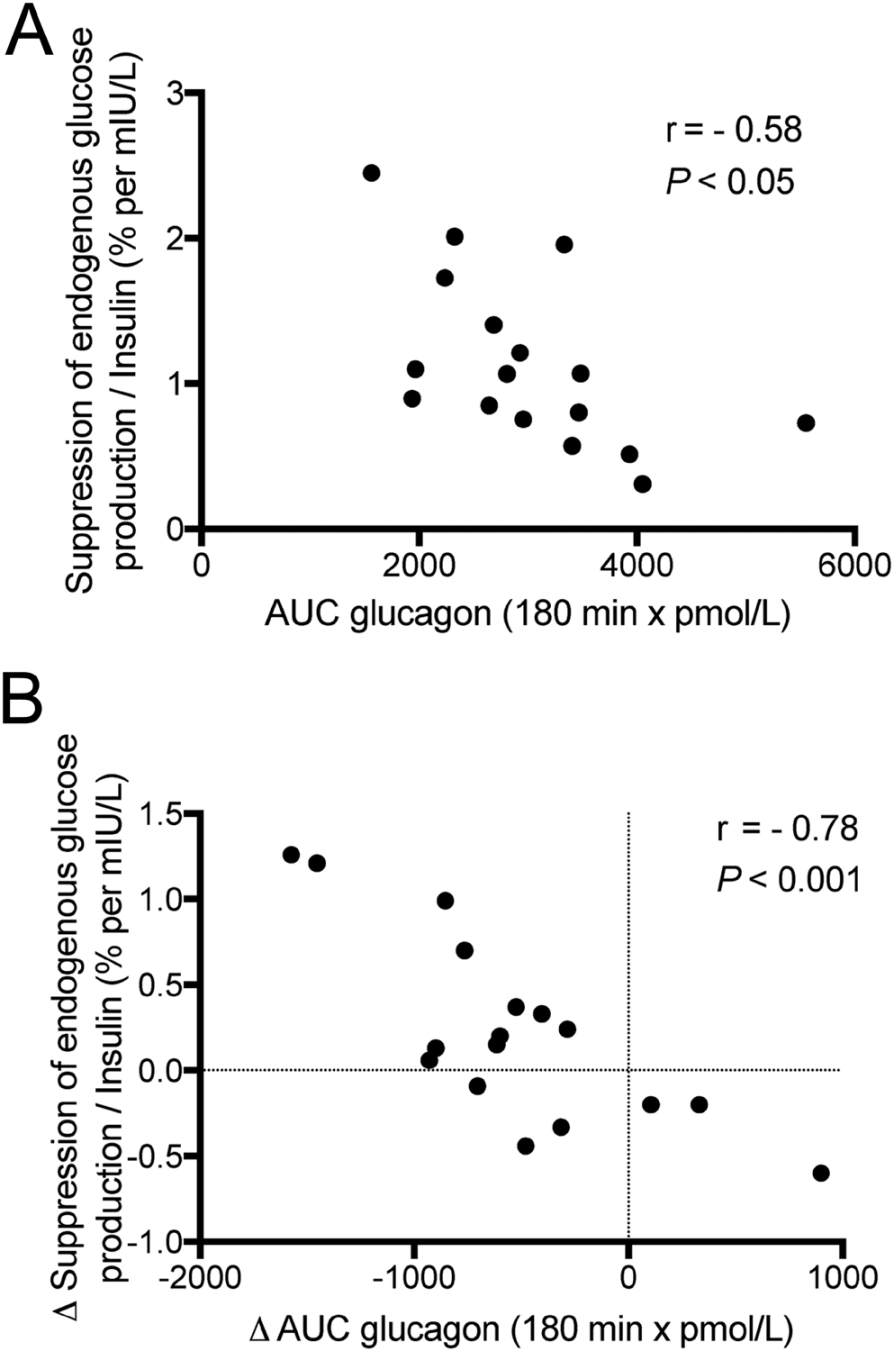
Association between postprandial glucagon and suppression of endogenous glucose production at baseline (A) and during 12 weeks of intervention with diet or diet and exercise (B). The correlation analyses were performed using Pearson correlation (r).

Analyzed with the multivariate model, we found a significant association between the postprandial increase of plasma glucagon from 0 to 60 min and the postprandial increase of the following plasma amino acids (ordered according to the strength of the association to glucagon): alanine, leucine, asparagine, glutamate, serine, tryptophan, methionine, valine, isoleucine, lysine, arginine, phenylalanine, threonine, glycine, and tyrosine (Fig. 2). Notably, glutamine was not associated with postprandial glucagon levels. Hepatic insulin sensitivity, measured as suppression of endogenous glucose production normalized by insulin, was negatively associated with the postprandial response of glucagon to the mixed meal (r = −0.58, *P* < 0.05; Fig. 3A). The postprandial glucagon response was positively associated with body weight (r_S_ = 0.49, *P* < 0.05).

### The liver-alpha-cell axis during 12 weeks of weight loss

The main results of the 12-week intervention on body composition, liver fat, and hepatic insulin sensitivity and the validation of the intervention have been published previously (14, 15). Regarding the intervention with the Paleolithic diet, both study groups similarly decreased their intake of carbohydrates, saturated fat and sodium and report compliance to the Paleolithic diet for 94% of their energy intake (14, 22). Median weight loss was similar (7 kg) in both intervention groups (Supplementary Table 3).

**Figure 4:**
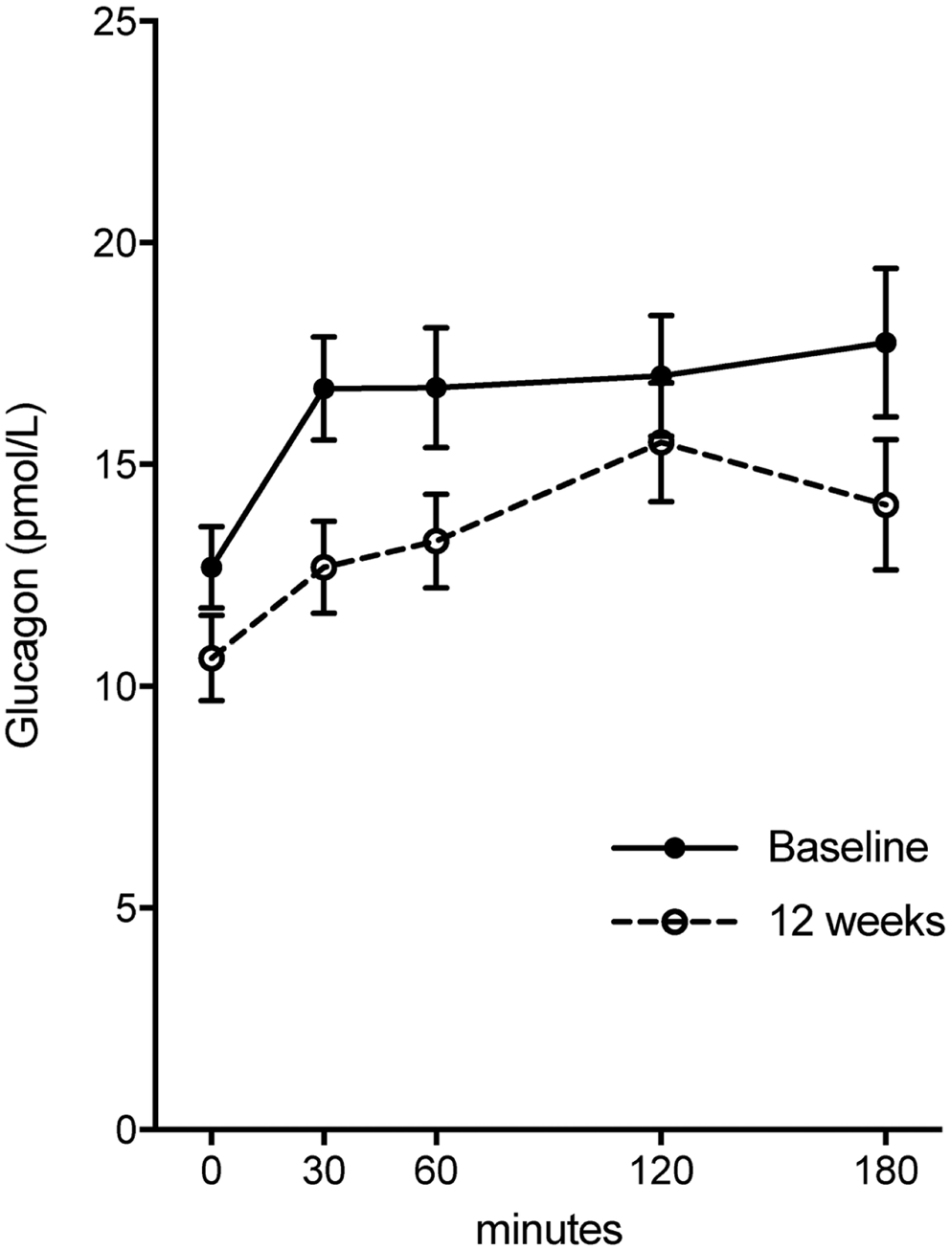
Postprandial plasma levels of glucagon during 12 weeks weight loss. Data are presented as mean ± s.e.m.

In the combined intervention group, fasting and postprandial glucagon decreased during 12 weeks of intervention (Fig. 4 and Table 2). In contrast, fasting and postprandial amino acids did not change after weight loss (Table 2).

**Table 2:** Effect of 12 weeks of weight loss intervention on weight, hepatic insulin sensitivity, and fasting and postprandial plasma levels of glucagon, amino acids, glucose, and insulin. Data are reported as the median (interquartile range) except for suppression of endogenous glucose production/insulin which is normally distributed and, therefore, depicted as mean ± s.d.; weight, liver fat, and suppression of endogenous glucose production reported in this table have been published previously (14, 15).

	Baseline	12 weeks
Weight, kg, *n* = 29	95.1 (83.3–103.2)	85.1 (76.8–97.6)^a^
Liver fat, %, *n* = 24	16.0 (11.2–26.2)	6.0 (1.7–12.9)^a^
Suppression of endogenous glucose production/insulin, % per mIU/L, *n* = 25	1.24 ± 0.53	1.49 ± 0.58^c^
Fasting glucagon, pmol/L, *n* = 22	12 (10–16)	9 (8–12)^c^
AUC glucagon, 180 min × pmol/L, *n* = 19	2955 (2325–3825)	2235 (1920–2700)^b^
Fasting amino acids, µmol/L, *n* = 12	2103 (1816–2365)	2036 (1912–2430)
AUC amino acids, 180 min × µmol/L, *n* = 12	429,856 (378,168–444,777)	402,751 (418,795–457,965)
Fasting glutamine, µmol/L, *n* = 13	479 (426–538)	497 (456–585)
AUC glutamine, 180 min × µmol/L, *n* = 12	88,415 (74,369–108,977)	89,734 (83,127–96,935)
Fasting alanine, µmol/L, *n* = 13	320 (257–391)	294 (246–357)
AUC alanine, 180 min × µmol/L, *n* = 13	69,578 (58,299–82,306)	67,689 (55,963–83,446)
Fasting glucose, mmol/L, *n* = 28	8.2 (7.0–9.5)	6.5 (6.0–7.5)^a^
AUC glucose, 180 min × mmol/L, *n* = 27	1456 (1254–1766)	1191 (1082–1317)^a^
Fasting insulin, mIU/L, *n* = 29	15.6 (10.5–24.5)	10.4 (6.7–15.2)^b^
AUC insulin, 180 min × mIU/L, *n* = 28	5495 (3347–7003)	4251 (2351–5587)^b^

^a^*P* < 0.001 for the intervention effect from baseline to 12 weeks; ^b^*P* < 0.01; ^c^*P* < 0.05.

AUC, area under the curve.

Weight loss was associated with decreasing fasting glucagon levels during the intervention (r_S_ = 0.45, *P* < 0.05). No correlation was found between the change in fasting glucagon during the intervention and the change in total fasting amino acids, fasting glutamine, or fasting alanine (r_S_ = 0.18, *P* = 0.61 and r_S_ = 0.13, *P* = 0.71 and r_S_ = 0.33, *P* = 0.32, respectively).

Weight loss correlated with decreasing postprandial glucagon levels during the intervention (r_S_ = 0.46, *P* < 0.05).

Hepatic insulin sensitivity measured as suppression of endogenous glucose production normalized for plasma insulin levels improved after weight loss (Table 2) (15). The improvement in hepatic insulin sensitivity was associated with decreased postprandial glucagon response to the mixed meal after the interventions (r = −0.78, *P* < 0.001; Fig. 3B).

### The liver-alpha-cell axis and liver fat

Liver fat content was not associated with glucagon, alanine, glutamine, or total amino acid levels in the fasted or postprandial state. Liver fat decreased during the 12-week intervention (Table 2), but this change was not associated with intervention effects on the liver-alpha-cell axis (data not shown).

### Effect of the 12-week intervention with either diet alone or diet combined with exercise and baseline characteristics of both intervention groups

Baseline characteristics for each intervention group are found in Supplementary Tables 2 and 3. At the study start, fasting glucose was higher and postprandial amino acids were lower in the diet and exercise group compared to the diet group (Supplementary Table 3).

Fasting glutamine significantly increased in the diet and exercise group (Supplementary Table 3; *P* < 0.05), without significant group differences over time.

Postprandial glucagon levels, after intake of the mixed meal, decreased by 22% in the diet group (*P* < 0.001) and by 18% in the diet and exercise group (*P* = 0.15; Supplementary Fig. 1 and Supplementary Table 3).

The postprandial alanine response decreased in the diet group only (group difference over time *P* < 0.05; Supplementary Fig. 2 and Supplementary Table 3). In contrast, postprandial glutamine was not changed by the diet only intervention but increased in the diet and exercise group (*P* < 0.05; Supplementary Fig. 2 and Supplementary Table 3). The postprandial levels of the remaining 18 amino acids during the intervention are reported in Supplementary Fig. 2.

## Discussion

The concept of a liver-alpha-cell axis implies that glucagon and amino acids are tightly regulated in a feedback loop (23). After the ingestion of a protein-rich meal, rising glucagon levels were not associated with glutamine but with several other amino acids including alanine. Moreover, hepatic insulin sensitivity was negatively associated with fasting and postprandial glucagon levels. The inverse association between hepatic insulin sensitivity and postprandial glucagon could be confirmed during weight loss. We conclude that postprandial glucagon secretion seems to be tightly related to hepatic insulin sensitivity.

Glucagon receptors are almost exclusively found in the liver (and in the kidney) where glucagon increases endogenous glucose production and ureagenesis (23). In response to a glucagon receptor antagonist, plasma amino acid levels rise within hours (24). It has, therefore, been proposed that glucagon plays an important role in acute amino acid metabolism (i.e. ureagenesis) in the liver (23). This regulatory cycle is thought to be important for maintaining normal amino acid and ammonia levels during protein abundance for example, after protein-rich meals.

In the fasted state, we found that individuals with higher total amino acid levels also had higher glucagon levels, in accordance with the concept of the liver-alpha-cell axis. In the fasted state, neither alanine nor glutamine was associated with glucagon. One possible explanation is that low glucagon levels in the fasting state are regulated by other factors including glucose levels (23). Thus, decreasing levels of glucose and amino acids during fasting will influence glucagon secretion in opposite directions in healthy individuals. However, in our patients with type 2 diabetes, it is more likely that the fasting glucose levels are maintained by (elevated) glucagon levels, as clearly demonstrated in experiments with glucagon receptor antagonists (25, 26). Notably, this study was only powered to investigate changes, for example, after a mixed meal and after weight loss and had, therefore, not enough participants to investigate any steady state, which would need a larger epidemiological sample.

Based on our results after the protein-rich meal, glutamine seems not to be a relevant signal molecule in the liver-alpha-cell axis. According to our postprandial analyses, alanine and 14 other amino acids could be the possible signal to the alpha cell to increase glucagon secretion. Based on earlier studies, some of these 15 amino acids are more likely to be a signal to the alpha cell than others. We propose alanine as a main signal molecule to the alpha cell because if administered intravenously, it is a potent stimulator of glucagon secretion (8). In the perfused mouse pancreas, alanine and five other amino acids but not glutamine were able to stimulate glucagon secretion (10). However, we also found a significant correlation between postprandial glucagon and the branched-chain amino acids leucine, isoleucine, and valine. Branched-chain amino acids are increased in plasma to a major extent after protein intake (27). These amino acids are metabolized in the muscle and not in the liver, and the glucagon receptor is expressed in the liver and not in muscle. Moreover, branched-chain amino acids do not stimulate glucagon secretion (3, 10) but are associated with insulin secretion in type 2 diabetes. Therefore, we regard the association between postprandial branched-chain amino acids and glucagon levels as related to beta-cell rather than alpha cell function.

Weight loss during our study was associated with decreasing fasting and postprandial glucagon levels. Our results are in line with two other studies that showed decreased fasting and postprandial glucagon levels after a mean weight loss of 10 kg in individuals with type 2 diabetes (28, 29). Thus, body weight may be one of the determinants of postprandial glucagon levels in type 2 diabetes.

Weight loss in our study decreased postprandial glucagon levels. Notably, we found a clear relationship between the ability of the weight loss to lower postprandial glucagon and the improved ability of the clamp to suppress endogenous glucose production. Already at the study start, hepatic insulin sensitivity was associated with postprandial glucagon levels. Thus, postprandial glucagon secretion seems to be tightly related to hepatic insulin sensitivity (30).

Nonalcoholic fatty liver disease has been proposed to impair hepatic sensitivity to glucagon signaling, causing higher levels of fasting amino acid and glucagon levels (23, 31). In our study, liver fat was not associated with amino acid levels or glucagon, neither at baseline nor during the intervention. The previous studies included participants with a higher degree of hepatic steatosis compared to our patients, which may explain these inconsistencies (31).

There were limitations to our study. First, this was a secondary analysis of outcomes not pre-defined before the study start. Therefore, analyses are exploratory, and results should be interpreted with caution. Secondly, the study population was small and amino acid analyses were only available for a subgroup in our study. Thirdly, the two intervention groups had different baseline values for fasting glucose and postprandial amino acids, which makes it difficult to compare intervention effects between study groups. Finally, we did not have a control group that was weight stable.

To conclude, several amino acids, notably alanine but not glutamine, could be the important signal to the alpha cell to increase glucagon secretion. Amino acids may be part of a feedback mechanism as glucagon increases endogenous glucose production and ureagenesis in the liver. Weight loss decreases postprandial glucagon levels, associated with decreased endogenous glucose production. This may indicate improved regulation of the liver-alpha-cell axis.

## Supplementary Material

Supplementary Materials

Supplementary Table 1 Amino acid content of one portion of the mixed meal

Supplementary Table 2 Baseline characteristics in both intervention groups

Supplementary Table 3 Effect of 12 weeks of intervention with diet or diet combined with exercise on weight, hepatic insulin sensitivity and fasting and postprandial plasma levels of glucose, insulin, glucagon and amino acids

Supplementary Table 4 Retention times (rt), MRM-transition stages monitored (precursor ion and product ions), collision energies and coefficients of variation (CV) of analyzed compounds.

Supplementary Fig. 1. Postprandial plasma levels of glucagon during 12 weeks weight loss intervention with diet (A) or diet and exercise (B). Data are presented as mean ± S.E.M.

Supplementary Fig. 2. Postprandial plasma levels of total amino acids, each amino acid separately, glucose and insulin during 12 weeks of weight loss intervention with diet or diet and exercise. Data are presented as mean ± S.E.M.

## Declaration of interest

The authors declare that there is no conflict of interest that could be perceived as prejudicing the impartiality of the research reported.

## Funding

This study was supported by grants from the Swedish Heart and Lung Foundation (20120450), King Gustav V and Queen Victoria’s Foundation, The Swedish Diabetes Research Foundation (2014-096), the County Council of Västerbotten (VLL-460481), and Umeå University, Sweden.

## Author contribution statement

Julia Otten: conceptualization, methodology, formal analysis, investigation, resources, writing – original draft, visualization. Andreas Stomby: conceptualization, methodology, investigation, resources, writing – original draft, project administration. Maria Waling: conceptualization, validation, investigation, writing – review and editing, supervision, project administration. Elin Chorell: methodology, validation, data curation, writing – review and editing, supervision. Mats Ryberg: conceptualization, methodology, resources, writing – review and editing. Michael Svensson: conceptualization, methodology, resources, writing – review and editing. Jens Juul Holst: conceptualization, validation, investigation, resources, writing – original draft, supervision. Tommy Olsson: conceptualization, methodology, writing – original draft, supervision, funding acquisition.

## References

[1] Muller WA Faloona GR Aguilar-Parada E Unger RH. Abnormal alpha-cell function in diabetes. Response to carbohydrate and protein ingestion. New England Journal of Medicine 1970 283 109–115. (10.1056/NEJM197007162830301) 4912452

[2] Toft-Nielsen MB Damholt MB Madsbad S Hilsted LM Hughes TE Michelsen BK Holst JJ. Determinants of the impaired secretion of glucagon-like peptide-1 in type 2 diabetic patients. Journal of Clinical Endocrinology and Metabolism 2001 86 3717–3723. (10.1210/jcem.86.8.7750) 11502801

[3] Rocha DM Faloona GR Unger RH. Glucagon-stimulating activity of 20 amino acids in dogs. Journal of Clinical Investigation 1972 51 2346–2351. (10.1172/JCI107046) PMC292401 4639019

[4] Holst JJ Wewer Albrechtsen NJ Pedersen J Knop FK. Glucagon and amino acids are linked in a mutual feedback cycle: the liver-alpha-cell axis. Diabetes 2017 66 235–240. (10.2337/db16-0994) 28108603

[5] Solloway MJ Madjidi A Gu C Eastham-Anderson J Clarke HJ Kljavin N Zavala-Solorio J Kates L Friedman B Brauer M Glucagon couples hepatic amino acid catabolism to mTOR-dependent regulation of alpha-cell mass. Cell Reports 2015 12 495–510. (10.1016/j.celrep.2015.06.034) 26166562

[6] Galsgaard KD Winther-Sorensen M Orskov C Kissow H Poulsen SS Vilstrup H Prehn C Adamski J Jepsen SL Hartmann B Disruption of glucagon receptor signaling causes hyperaminoacidemia exposing a possible liver – alpha-cell axis. American Journal of Physiology: Endocrinology and Metabolism 2018 314 E93–E103. (10.1152/ajpendo.00198.2017) 28978545 PMC6048389

[7] Dean ED Li M Prasad N Wisniewski SN Von Deylen A Spaeth J Maddison L Botros A Sedgeman LR Bozadjieva N Interrupted glucagon signaling reveals hepatic alpha cell axis and role for L-glutamine in alpha cell proliferation. Cell Metabolism 2017 25 1362.e5–1373.e5. (10.1016/j.cmet.2017.05.011) 28591638 PMC5572896

[8] Muller WA Faloona GR Unger RH. The effect of alanine on glucagon secretion. Journal of Clinical Investigation 1971 50 2215–2218. (10.1172/JCI106716) PMC292156 5116210

[9] Larger E Wewer Albrechtsen NJ Hansen LH Gelling RW Capeau J Deacon CF Madsen OD Yakushiji F De Meyts P Holst JJ Pancreatic alpha-cell hyperplasia and hyperglucagonemia due to a glucagon receptor splice mutation. Endocrinology, Diabetes and Metabolism Case Reports 2016 2016 16-0081. (10.1530/EDM-16-0081) PMC5118975 27933176

[10] Galsgaard KD Jepsen SL Kjeldsen SAS Pedersen J Wewer Albrechtsen NJ Holst JJ. Alanine, arginine, cysteine, and proline, but not glutamine, are substrates for, and acute mediators of, the liver-alpha-cell axis in female mice. American Journal of Physiology: Endocrinology and Metabolism 2020 318 E920–E929. (10.1152/ajpendo.00459.2019) 32255678

[11] Stancakova A Civelek M Saleem NK Soininen P Kangas AJ Cederberg H Paananen J Pihlajamaki J Bonnycastle LL Morken MA Hyperglycemia and a common variant of GCKR are associated with the levels of eight amino acids in 9369 Finnish men. Diabetes 2012 61 1895–1902. (10.2337/db11-1378) 22553379 PMC3379649

[12] Gonzalez-Franquesa A Burkart AM Isganaitis E Patti ME. What have metabolomics approaches taught us about type 2 diabetes? Current Diabetes Reports 2016 16 74. (10.1007/s11892-016-0763-1) PMC5441387 27319324

[13] Lim EL Hollingsworth KG Aribisala BS Chen MJ Mathers JC Taylor R. Reversal of type 2 diabetes: normalisation of beta cell function in association with decreased pancreas and liver triacylglycerol. Diabetologia 2011 54 2506–2514. (10.1007/s00125-011-2204-7) 21656330 PMC3168743

[14] Otten J Stomby A Waling M Isaksson A Tellstrom A Lundin-Olsson L Brage S Ryberg M Svensson M Olsson T. Benefits of a paleolithic diet with and without supervised exercise on fat mass, insulin sensitivity, and glycemic control: a randomized controlled trial in individuals with type 2 diabetes. Diabetes/Metabolism Research and Reviews 2017 33. (10.1002/dmrr.2828) PMC5402870 27235022

[15] Otten J Stomby A Waling M Isaksson A Soderstrom I Ryberg M Svensson M Hauksson J Olsson T. A heterogeneous response of liver and skeletal muscle fat to the combination of a paleolithic diet and exercise in obese individuals with type 2 diabetes: a randomised controlled trial. Diabetologia 2018 61 1548–1559. (10.1007/s00125-018-4618-y) 29696296 PMC6445456

[16] Wycherley TP Noakes M Clifton PM Cleanthous X Keogh JB Brinkworth GD. A high-protein diet with resistance exercise training improves weight loss and body composition in overweight and obese patients with type 2 diabetes. Diabetes Care 2010 33 969–976. (10.2337/dc09-1974) 20150293 PMC2858200

[17] Reaven GM Chen YD Golay A Swislocki AL Jaspan JB. Documentation of hyperglucagonemia throughout the day in nonobese and obese patients with noninsulin-dependent diabetes mellitus. Journal of Clinical Endocrinology and Metabolism 1987 64 106–110. (10.1210/jcem-64-1-106) 3536980

[18] Knop FK Vilsboll T Madsbad S Holst JJ Krarup T. Inappropriate suppression of glucagon during OGTT but not during isoglycaemic i.v. glucose infusion contributes to the reduced incretin effect in type 2 diabetes mellitus. Diabetologia 2007 50 797–805. (10.1007/s00125-006-0566-z) 17225124

[19] Jonsson P Wuolikainen A Thysell E Chorell E Stattin P Wikstrom P Antti H. Constrained randomization and multivariate effect projections improve information extraction and biomarker pattern discovery in metabolomics studies involving dependent samples. Metabolomics 2015 11 1667–1678. (10.1007/s11306-015-0818-3) 26491420 PMC4605978

[20] Eriksson L Trygg J Wold S. CV-ANOVA for significance testing of PLS and OPLS® models. Journal of Chemometrics 2008 22 594–600. (10.1002/cem.1187)

[21] Efron B Gong G. A leisurely look at the bootstrap, the jackknife, and cross-validation. American Statistician 1983 37 36–48. (10.1080/00031305.1983.10483087)

[22] Martensson A Stomby A Tellstrom A Ryberg M Waling M Otten J. Using a paleo ratio to assess adherence to paleolithic dietary recommendations in a randomized controlled trial of individuals with type 2 diabetes. Nutrients 2021 13 969. (10.3390/nu13030969) 33802738 PMC8002510

[23] Wewer Albrechtsen NJ Pedersen J Galsgaard KD Winther-Sorensen M Suppli MP Janah L Gromada J Vilstrup H Knop FK Holst JJ. The liver-alpha-cell axis and Type 2 diabetes. Endocrine Reviews 2019 40 1353–1366. (10.1210/er.2018-00251) 30920583

[24] Haedersdal S Wewer Albrechtsen NJ Lund AB Galsgaard KD Winther-Sorensen M Holst JjJ Knop FK Vilsboll T. Glucagon receptor antagonism increases plasma amino acids and glucagon. Diabetes 2019 68 1952-P.(10.2337/db19-1952-P)

[25] Kazda CM Ding Y Kelly RP Garhyan P Shi C Lim CN Fu H Watson DE Lewin AJ Landschulz WH Evaluation of efficacy and safety of the glucagon receptor antagonist LY2409021 in patients with type 2 diabetes: 12- and 24-week phase 2 studies. Diabetes Care 2016 39 1241–1249. (10.2337/dc15-1643) 26681715

[26] Haedersdal S Lund AB Maagensen H Nielsen-Hannerup E Holst JjJ Knop FK Vilsboll T. Individual and combined glucose-lowering effects of glucagon receptor antagonism and sodium-glucose cotransporter 2 inhibition. Diabetes 2018 67 1942-P. (10.2337/db18-1942-P)

[27] Ang T Bruce CR Kowalski GM. Postprandial aminogenic insulin and glucagon secretion can stimulate glucose flux in humans. Diabetes 2019 68 939–946. (10.2337/db18-1138) 30833465

[28] Laferrere B Teixeira J McGinty J Tran H Egger JR Colarusso A Kovack B Bawa B Koshy N Lee H Effect of weight loss by gastric bypass surgery versus hypocaloric diet on glucose and incretin levels in patients with type 2 diabetes. Journal of Clinical Endocrinology and Metabolism 2008 93 2479–2485. (10.1210/jc.2007-2851) 18430778 PMC2453054

[29] Marfella R Barbieri M Ruggiero R Rizzo MR Grella R Mozzillo AL Docimo L Paolisso G. Bariatric surgery reduces oxidative stress by blunting 24-h acute glucose fluctuations in type 2 diabetic obese patients. Diabetes Care 2010 33 287–289. (10.2337/dc09-1343) 19889803 PMC2809267

[30] Wewer Albrechtsen NJ Faerch K Jensen TM Witte DR Pedersen J Mahendran Y Jonsson AE Galsgaard KD Winther-Sorensen M Torekov SS Evidence of a liver-alpha cell axis in humans: hepatic insulin resistance attenuates relationship between fasting plasma glucagon and glucagonotropic amino acids. Diabetologia 2018 61 671–680. (10.1007/s00125-017-4535-5) 29305624

[31] Wewer Albrechtsen NJ Junker AE Christensen M Haedersdal S Wibrand F Lund AM Galsgaard KD Holst JJ Knop FK Vilsboll T. Hyperglucagonemia correlates with plasma levels of non-branched chained amino acids in patients with liver disease independent of type 2 diabetes. American Journal of Physiology: Gastrointestinal and Liver Physiology 2017 314 G91–G96. (10.1152/ajpgi.00216.2017) 28971838

