# Supplementary material for: The liver-alpha-cell axis after a mixed meal and during weight loss in type 2 diabetes
Source: Endocr Connect. 2021 Aug 11;10(9):1101–10. doi: 10.1530/EC-21-0171 (PMC8494406; doi:10.1530/EC-21-0171)
Supplement: Supplementary Materials [file supplementary_material.pdf]

Supplementary Methods Analysis of plasma amino acids

Standards and calibration curve

Amino acid standards (alanine, arginine, aspartic acid, cysteine, glutamic acid, glycine, histidine, isoleucine, leucine, lysine, methionine, phenylalanine, proline, serine, threonine, tyrosine, valine, glutamine, asparagine, GABA, citrulline, ornithine, taurine, tryptophan, 5-HTP, kynurenine and norvaline) were purchased from Sigma (St. Louis, MO, USA). Isotopically-labeled amino acid standards (alanine ($^{13}C_3$, ^{15}N), arginine ($^{13}C_6$, $^{15}N_4$), aspartic acid ($^{13}C_4$, ^{15}N), cystine ($^{13}C_6$, $^{15}N_2$), glutamic acid ($^{13}C_5$, ^{15}N), glycine ($^{13}C_2$, ^{15}N), histidine ($^{13}C_6$, $^{15}N_3$), isoleucine ($^{13}C_6$, ^{15}N), leucine ($^{13}C_6$, ^{15}N), lysine ($^{13}C_6$, $^{15}N_2$), methionine ($^{13}C_5$, ^{15}N), phenylalanine ($^{13}C_9$, ^{15}N), proline ($^{13}C_5$, ^{15}N), serine ($^{13}C_3$, ^{15}N), threonine ($^{13}C_4$, ^{15}N), tyrosine ($^{13}C_9$, ^{15}N), valine ($^{13}C_5$, ^{15}N), Citrulline (d4), glutamine ($^{13}C_5$), asparagine($^{13}C_4$), tryptophan (d8) were obtained from Cambridge Isotope Laboratories (Andover, MA, USA). Stock solutions of each compound were prepared at a concentration of 500 ng/µL and stored at -80ºC. A 10-point calibration curve (0.01–100 pmol/µL) was prepared by serial dilutions. A volume of 10 µL of each calibration point was evaporated to dryness. MS-grade formic acid was purchased from Sigma-Aldrich (St Louis, MO, USA) and high-performance liquid chromatography-grade acetonitrile from Fisher Scientific (Fair Lawn, NJ, USA).

Extraction of amino acids in plasma

Amino acids were extracted by mixing 100 µL plasma samples with 900 µL 90:10 (v/v) methanol:water solution containing norvaline at 2.2 pmol/µL as an internal standard. Each sample was extracted for 2 min using a mixer mill, incubated in the freezer for 2 h, and centrifuged at 4ºC, 14000 rpm for 10 min. Then 25 µL of supernatant was transferred to microvials and evaporated to dryness in a speed-vac concentrator. The samples were stored at -80°C until analysis.

Amino acid derivatization with AccQ.Tag

Extracted samples were derivatized by AccQ-Tag™ (Waters) according to the manufacturer’s instructions. Briefly, the dried extracts were re-suspended in 20 µL of 20 mM HCl, and 60 µL AccQ•Tag Ultra borate buffer spiked with all isotopically labeled internal standards at a final concentration of 0.833 pmol/µL was added to each sample. Finally, 20 µL freshly prepared AccQ•Tag derivatization solution was added and the samples were immediately vortexed for 10 s. The dried calibration curves were prepared in a similar way using the same spiked ultra borate buffer. Samples were kept at room temperature for 30 min followed by 10 min at 55ºC. For each batch, quality control samples, and procedure, blanks were included.

Amino acid quantification by LC-electrospray ionization-MS/MS

Derivatized samples were analyzed using the 1290 Infinitely system from Agilent Technologies, consisting of the G4220A binary pump, G1316C thermostated column compartment, and G4226A autosampler with G1330B autosampler thermostat coupled to the Agilent 6460 triple quadrupole mass spectrometer equipped with a jet stream electrospray source operating in positive ion mode. Separation was achieved injecting 1 µL of each sample

onto a BEH C_{18} 2.1x100 mm, 1.7 µm column (Waters) held at 50ºC in a column oven. The gradient eluents used were H_2O 0.1% formic acid (A) and acetonitrile 0.1% formic acid (B) with a flow rate of 500 µL/min. The initial conditions consisted of 0% B, and the following gradient was used with linear increments: 0.54–3.50 min (0.1–9.1% B), 3.50–7.0 (9.1–17.0% B), 7.0–8.0 (17.0–19.70% B), 8.0–8.5 (19.7% B), 8.5–9.0 (19.7–21.2% B), 9.0–10.0 (21.2–59.6% B), 10.0–11.0 (59.6–95.0% B), 11.0–11.5 (95.0% B), 11.5–15.0 (0% B). From 13.0 min to 14.8 min, the flow rate was set at 800 µL/min for a faster equilibration of the column. Multiple reaction monitoring (MRM) transitions for the derivatized amino acids were optimized using MassHunter MS Optimizer software (Agilent Technologies Inc., Santa Clara, CA, USA). The fragmentor voltage was set at 380 V, the cell accelerator voltage was 7 V, and the collision energies ranged from 14 V to 45 V; nitrogen was used as the collision gas (Supplementary Table 4). The data were quantified using MassHunter™ Quantitation software B08.00 (Agilent Technologies, USA) and the amount of each amino acid was calculated based on the calibration curves.

MS parameters

Jet stream gas temperature was 290°C with a gas flow of 11 L/min, sheath gas temperature 325°C, and sheath gas flow of 12 L/min. The nebulizer pressure was set to 20 psi and the capillary voltage was set at 4 kV. The QqQ was run in dynamic MRM mode with 2 min retention time windows and 500 ms cycle scans (Supplementary Table 4).
