# Supplementary material for: The liver-alpha-cell axis after a mixed meal and during weight loss in type 2 diabetes
Source: Endocr Connect. 2021 Aug 11;10(9):1101–10. doi: 10.1530/EC-21-0171 (PMC8494406; doi:10.1530/EC-21-0171)
Supplement: Supplementary Table 1 Amino acid content of one portion of the mixed meal [file supplementary_table_1.pdf]

Supplementary Table 1 Amino acid content of one portion of the mixed meal

	Amino acid content in one portion of the mixed meal (mg)	Amino acid content in % of protein content of the mixed meal
Glutamine/glutamate	4,239	15.7
Asparagine/aspartate	2,622	9.7
Leucine	2,165	8.0
Lysine	2,136	7.9
Arginine	1,862	6.9
Valine	1,573	5.8
Alanine	1,487	5.5
Serine	1,399	5.2
Isoleucine	1,351	5.0
Phenylalanine	1,282	4.8
Glycine	1,237	4.6
Proline	1,237	4.6
Threonine	1,130	4.2
Tyrosine	948	3.5
Histidine	788	2.9
Methionine	742	2.8
Cysteine	395	1.5
Tryptophan	339	1.3

The amino acid content of the mixed meal is calculated based on the following references: 1) U.S. Department of Agriculture, Human Nutrition Information Service. Composition of Foods 8:1-12. US Gov. Printing Office, Washington DC 1976-1984. 2) FAO. Amino acid content of foods and biological data on proteins. FAO Nutrition studies 24. Rome, 1970. 3) Söndergaard H. Aminosyreinholdet i danske levnedsmidler. Statens Levnedsmiddelinstitut, Publikation nr 98, November 1984. 4) Svanberg U, Gebre-Mehdin M, Ljungquist B, Olsson M. Breast Milk composition Ethiopian and Swedish mothers. III Amino acids and other nitrogenous substances. AM J Clin Nutr 1977;30:499-507
