# Supplementary material for: The liver-alpha-cell axis after a mixed meal and during weight loss in type 2 diabetes
Source: Endocr Connect. 2021 Aug 11;10(9):1101–10. doi: 10.1530/EC-21-0171 (PMC8494406; doi:10.1530/EC-21-0171)
Supplement: Supplementary Table 2 Baseline characteristics in both intervention groups [file supplementary_table_2.pdf]

Supplementary Table 2 Baseline characteristics in both intervention groups

	Diet group	Diet + Exercise group
n (male/female)	15 (10/5)	14 (9/5)
Age, years	60 (53–64)	61 (58–66)
BMI, kg/m^2	31.4 (29.1–33.4)	31.7 (29.0–36.4)
Diabetes duration, years	3 (1–5)	6 (1–8)
HbA1c, mmol/mol (ref 31-47)	53 (48–57)	57 (51–64)
HbA1c, % (ref 5.0-7.7)	7.0 (6.5–7.4)	7.4 (6.8–8.0)
Systolic blood pressure, mmHg	135 (127–148)	132 (122–143)
Diastolic blood pressure, mmHg	86 (82–94)	82 (74–91)
Total cholesterol, mmol/L (ref 3.9-7.8)	4.2 (3.4–4.7)	4.3 (4.1–4.9)
LDL, mmol/L (ref 2.0-5.3)	2.1 (1.8–2.7)	2.4 (2.0–3.0)
Triglycerides, mmol/L (ref <2.6)	2.1 (1.4–2.9)	1.7 (1.1–2.4)
Metformin therapy	10	10
Statin therapy	6	8

Data are reported as the median (interquartile range). There were no significant differences in any of the baseline characteristics between intervention groups.
