# Supplementary material for: The liver-alpha-cell axis after a mixed meal and during weight loss in type 2 diabetes
Source: Endocr Connect. 2021 Aug 11;10(9):1101–10. doi: 10.1530/EC-21-0171 (PMC8494406; doi:10.1530/EC-21-0171)
Supplement: Supplementary Table 3 Effect of 12 weeks of intervention with diet or diet combined with exercise on weight, hepatic insulin sensitivity and fasting and postprandial plasma levels of glucose, insulin, glucagon and amino acids [file supplementary_table_3.pdf]

Supplementary Table 3 Effect of 12 weeks of intervention with diet or diet combined with exercise on weight, hepatic insulin sensitivity and fasting and postprandial plasma levels of glucose, insulin, glucagon and amino acids

	Diet	Diet + Exercise
Weight, kg, n=15/14		
Baseline	90.0 (83.3 – 101.9)	97.3 (83.4 – 113.9)
Change 0-12 weeks	-7.1 (-10.1, -6.1)***	-7.1 (-9.5, -5.8)***
Liver fat, % n=12/12		
Baseline	20.5 (13.6 – 27.8)	13.0 (10.8 – 25.1)
Change 0-12 weeks	-12,8 (-17.1, -9.2)**	-4.2 (-11.8, 4.3)[a]
Suppression of endogenous glucose production/Insulin, % per mIU/L, n=12/13		
Baseline	0.98 (0.77 – 1.36)	1.51 (0.97 – 1.74)
Change 0-12 weeks	0.22 (-0.03, 0.91)*	0.06 (-0.45, 0.55)
Fasting glucagon, pmol/L, n=12/10		
Baseline	13 (10-17)	11 (9-14)
Change 0-12 weeks	-3 (-4 ,0)	-1 (-6, 1)
AUC glucagon, 180 min x pmol/L, n=12/7		
Baseline	3,124 ± 1066	3,077 ± 782
Change 0-12 weeks	-673 ± 483***	-551 ± 876
Fasting amino acids, μmol/L, n=5/7		
Baseline	2,413 (1,794 – 2,548)	1,970 (1,814 – 2,208)
Change 0-12 weeks	-103 (-415, 536)	30 (-131, 663)
AUC amino acids, 180 min x μmol/L, n=5/7		
Baseline	444,931 (430,421 – 498,259)	386,851 (374,898 – 440,368)[a]
Change 0-12 weeks	-3,091 (-83,111, 11,881)	13,884 (750, 83,807)*
Fasting glutamine, μmol/L, n=5/7		
Baseline	531 (426 – 652)	468 (401 – 518)
Change 0-12 weeks	-37 (-183, 95)	95 (-5, 128)*
AUC glutamine, 180 min x μmol/L, n=5/7		
Baseline	98,195 (85,065 – 113,322)	84,964 (69,692 – 90,177)
Change 0-12 weeks	-6,099 (-26,895, 5,374)	15,072 (6,599, 24,254)*[a]
Fasting alanine, μmol/L, n=5/8		
Baseline	389 (253 – 504)	294 (255 – 355)
Change 0-12 weeks	-26 (-155, 39)	4 (-67, 101)
AUC alanine, 180 min x μmol/L, n=5/8		
Baseline	80,050 (68,173 – 105,031)	64,442 (54,404 – 72,449)
Change 0-12 weeks	-9,043 (-49,993, -4,489)*	4,741 (-4,773, 15,907)[a]
Fasting glucose, mmol/L, n=14/14		
Baseline	8.0 (6.5 – 8.3)	9.1 (7.7 – 12.0)[a]
Change 0-12 weeks	-1.0 (-2.2, -0.4)	-2.2 (-3.7, -0.7)**
AUC glucose, 180 min x mmol/L, n=14/13		
Baseline	1,403 (1,206 – 1,601)	1,715 (1,307 – 2,020)
Change 0-12 weeks	-167 (-350, -8)*	-432 (-683, -140)**
Fasting insulin, mIU/L, n=15/14		
Baseline	17.5 (9.7 – 28.3)	14.3 (11.8 – 21.5)
Change 0-12 weeks	-3.4 (-15.7, 5.5)	-4.1 (-8.1, -1.4)***
AUC insulin, 180 min x mIU/L, n=15/13		
Baseline	5,500 (2,855 – 7,159)	5,228 (3,474 – 6,898)
Change 0-12 weeks	-1,099 (-2,898, 148)	-1,277 (-2,267, 16)

Data are reported as the median (interquartile range) except for AUC glucagon which was normally distributed and therefore presented as mean ± standard deviation. Weight, liver fat and suppression of endogenous glucose production reported in this table have been published previously (1, 2); *P<0.05, **P<0.01, ***P<0.001 for the change over time from baseline to 12 weeks within the group; [a]P<0.05 between the diet group and the diet + exercise group; AUC, area under the curve;

References

1 Otten J, Stomby A, Waling M, Isaksson A, Tellstrom A, Lundin-Olsson L, Brage S, Ryberg M, Svensson M & Olsson T. Benefits of a Paleolithic diet with and without supervised exercise on fat mass, insulin sensitivity, and glycemic control: a randomized controlled trial in individuals with type 2 diabetes. *Diabetes Metab Res Rev* 2017 **33**.

2 Otten J, Stomby A, Waling M, Isaksson A, Soderstrom I, Ryberg M, Svensson M, Hauksson J & Olsson T. A heterogeneous response of liver and skeletal muscle fat to the combination of a Paleolithic diet and exercise in obese individuals with type 2 diabetes: a randomised controlled trial. *Diabetologia* 2018 **61** 1548-1559.
