# Supplementary material for: The liver-alpha-cell axis after a mixed meal and during weight loss in type 2 diabetes
Source: Endocr Connect. 2021 Aug 11;10(9):1101–10. doi: 10.1530/EC-21-0171 (PMC8494406; doi:10.1530/EC-21-0171)
Supplement: Supplementary Table 4 Retention times (rt), MRM-transition stages monitored (precursor ion and product ions), collision energies and coefficients of variation (CV) of analyzed compounds. [file supplementary_table_4.pdf]

Supplementary Table 4 Retention times (rt), MRM-transition stages monitored (precursor ion and product ions), collision energies and coefficients of variation (CV) of analyzed compounds.

Compounds	MRM transition		rt (min)	Collision Energy (V)	CV (%)
	Precursor Ion	Product Ion			
alanine	260.1	171	4.1	14	4.3
arginine	345.1	171	2.8	35	11.1
aspartic acid	304.1	171	3.4	18	8.0
glutamic acid	318.1	171	3.6	22	5.6
glycine	246.1	171	3.1	18	3.0
histidine	326.1	171	2.6	26	10.6
isoleucine	302.1	171	7.8	18	11.9
leucine	302.1	171	7.6	18	16.8
lysine	487.2	171	5.1	26	7.7
methionine	320.1	171	6.0	22	9.1
phenylalanine	336.1	171	8.1	18	7.6
proline	286.1	171	4.4	14	8.4
serine	276.1	171	3.1	14	9.2
threonine	290.1	171	3.7	18	15.5
tyrosine	352.1	171	5.7	18	8.8
valine	288.1	171	6.1	18	10.5
citrulline	346.2	171	3.4	30	11.6
glutamine	317.1	171	3.0	22	19.7
asparagine	303.1	171	2.8	18	10.4
tryptophan	375.2	171	8.4	26	10.9
Internal standards					
alanine (13C3, 15N)	264.07	171	4.1	14	
arginine (13C6, 15N4)	355.1	171	2.8	35	
aspartic acid (13C4, 15N)	309.07	171	3.3	18	
cystine (13C6, 15N2)	589	171	5.2	25	
glutamic acid (13C5, 15N)	324.09	171	3.5	22	
glycine (13C2, 15N)	249.05	171	3.1	18	
histidine (13C6, 15N3)	335	171	2.6	26	
isoleucine (13C6, 15N)	309.12	171	7.9	18	
leucine (13C6, 15N)	309.12	171	7.6	18	
lysine (13C6, 15N2)	495.1	171	5.2	26	
methionine (13C5, 15N)	326.17	171	6.0	22	
phenylalanine (13C9, 15N)	346	171	8.2	18	
proline (13C5, 15N)	292.09	171	4.4	14	

serine (13C3, 15N)	280.6	171	3.0	14	
threonine (13C4, 15N)	295.08	171	3.7	18	
tyrosine (13C9, 15N)	362.12	171	5.7	18	
valine (13C5, 15N)	294.1	171	6.1	18	
Citrulline (d4)	350.1	171	3.4	30	
glutamine (13C5)	322.1	171	3.1	22	
asparagine(13C4)	307.1	171	2.8	18	
tryptophan (d8)	383.2	171	8.3	26	
