# Supplementary material for: The liver-alpha-cell axis after a mixed meal and during weight loss in type 2 diabetes
Source: Endocr Connect. 2021 Aug 11;10(9):1101–10. doi: 10.1530/EC-21-0171 (PMC8494406; doi:10.1530/EC-21-0171)

Supplementary Fig. 1. Postprandial plasma levels of glucagon during 12 weeks weight loss intervention with diet (A) or diet and exercise (B). Data are presented as mean ± S.E.M.

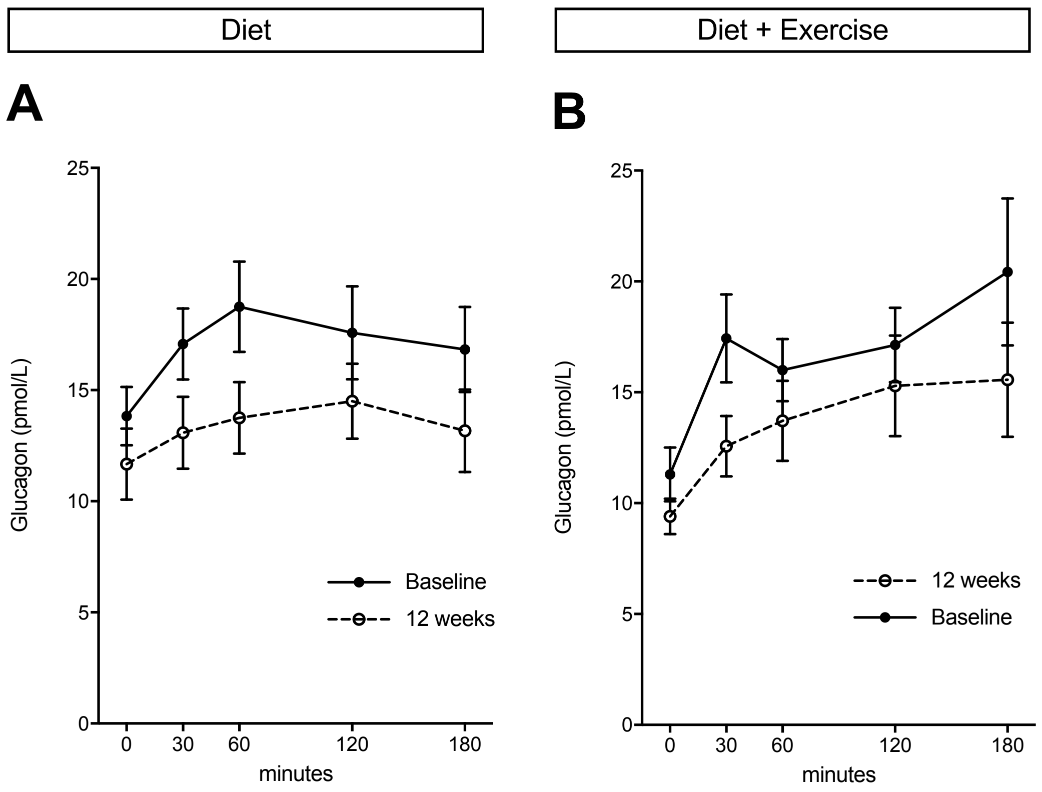

Supplement: Supplementary Fig. 1. Postprandial plasma levels of glucagon during 12 weeks weight loss intervention with diet (A) or diet and exercise (B). Data are presented as mean ± S.E.M. [file supplementary_figure_1.pdf]
