# Supplementary material for: The liver-alpha-cell axis after a mixed meal and during weight loss in type 2 diabetes
Source: Endocr Connect. 2021 Aug 11;10(9):1101–10. doi: 10.1530/EC-21-0171 (PMC8494406; doi:10.1530/EC-21-0171)
Supplement: Supplementary Fig. 2. Postprandial plasma levels of total amino acids, each amino acid separately, glucose and insulin during 12 weeks of weight loss intervention with diet or diet and exercise. Data are presented as mean ± S.E.M. [file supplementary_figure_2.pdf]

Supplementary Fig. 2. Postprandial plasma levels of total amino acids, each amino acid separately, glucose and insulin during 12 weeks of weight loss intervention with diet or diet and exercise. Data are presented as mean ± S.E.M.

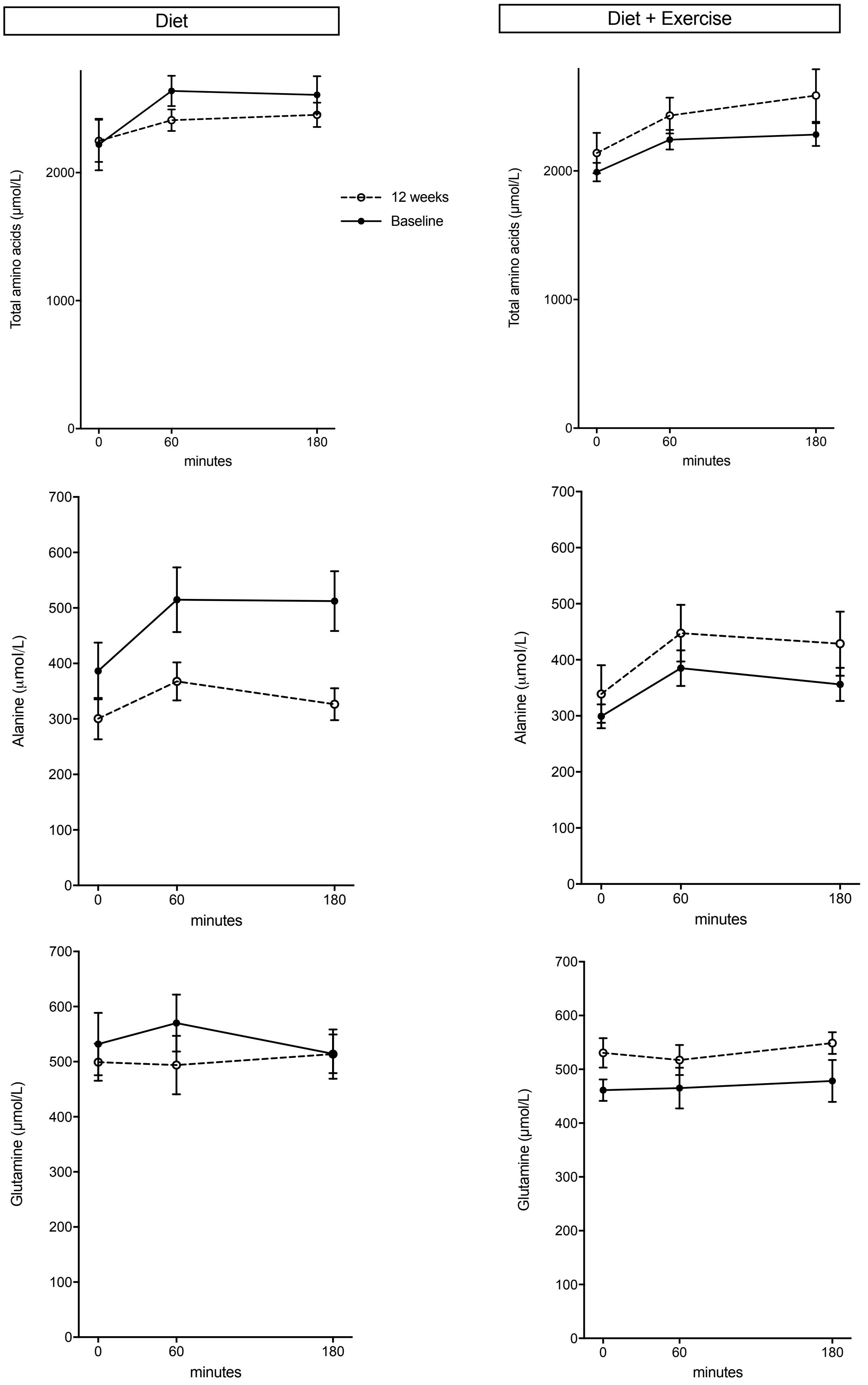

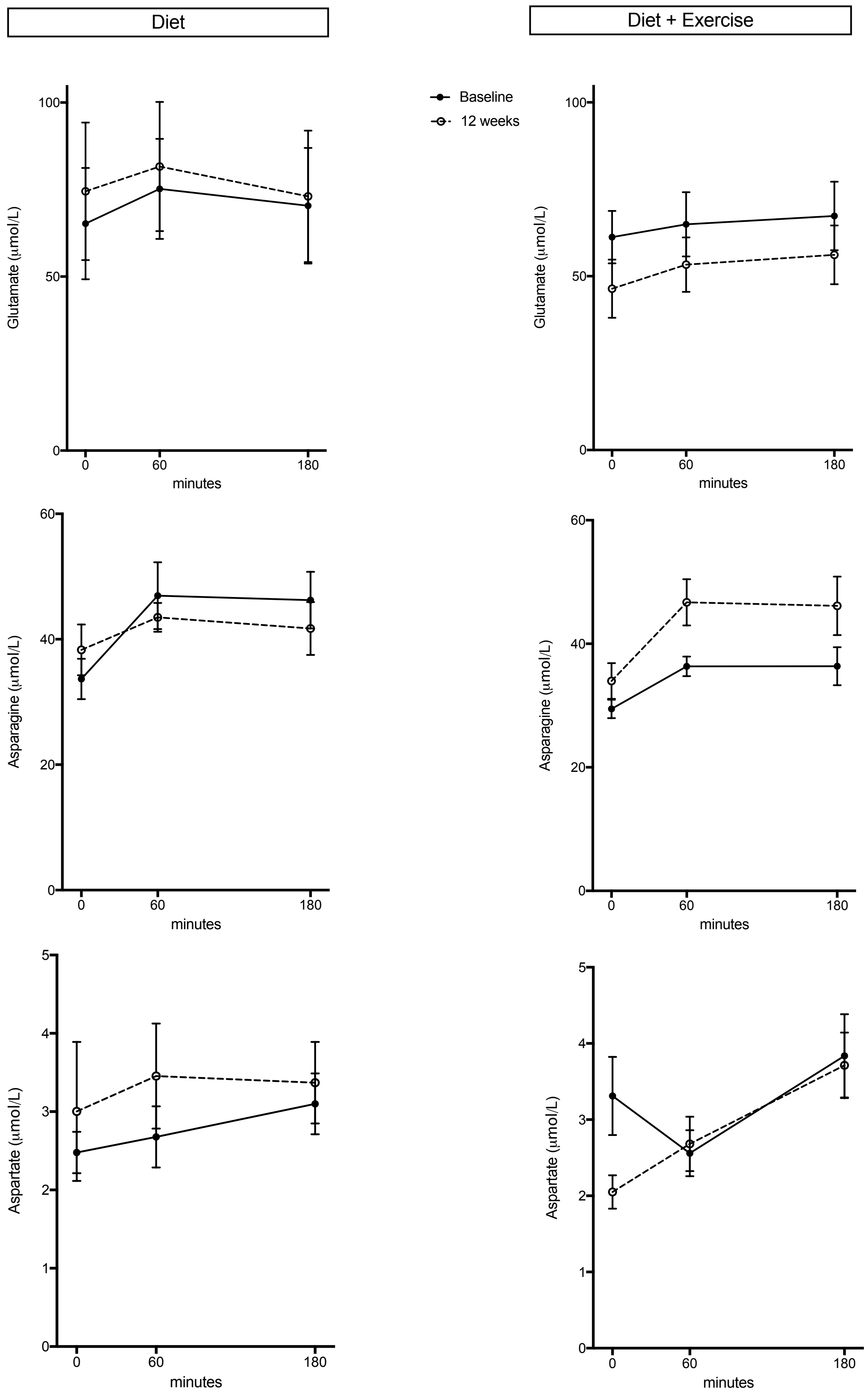

Diet
Diet + Exercise
Baseline
12 weeks
Glutamate (µmol/L)
Asparagine (µmol/L)
Aspartate (µmol/L)
minutes
0
60
180

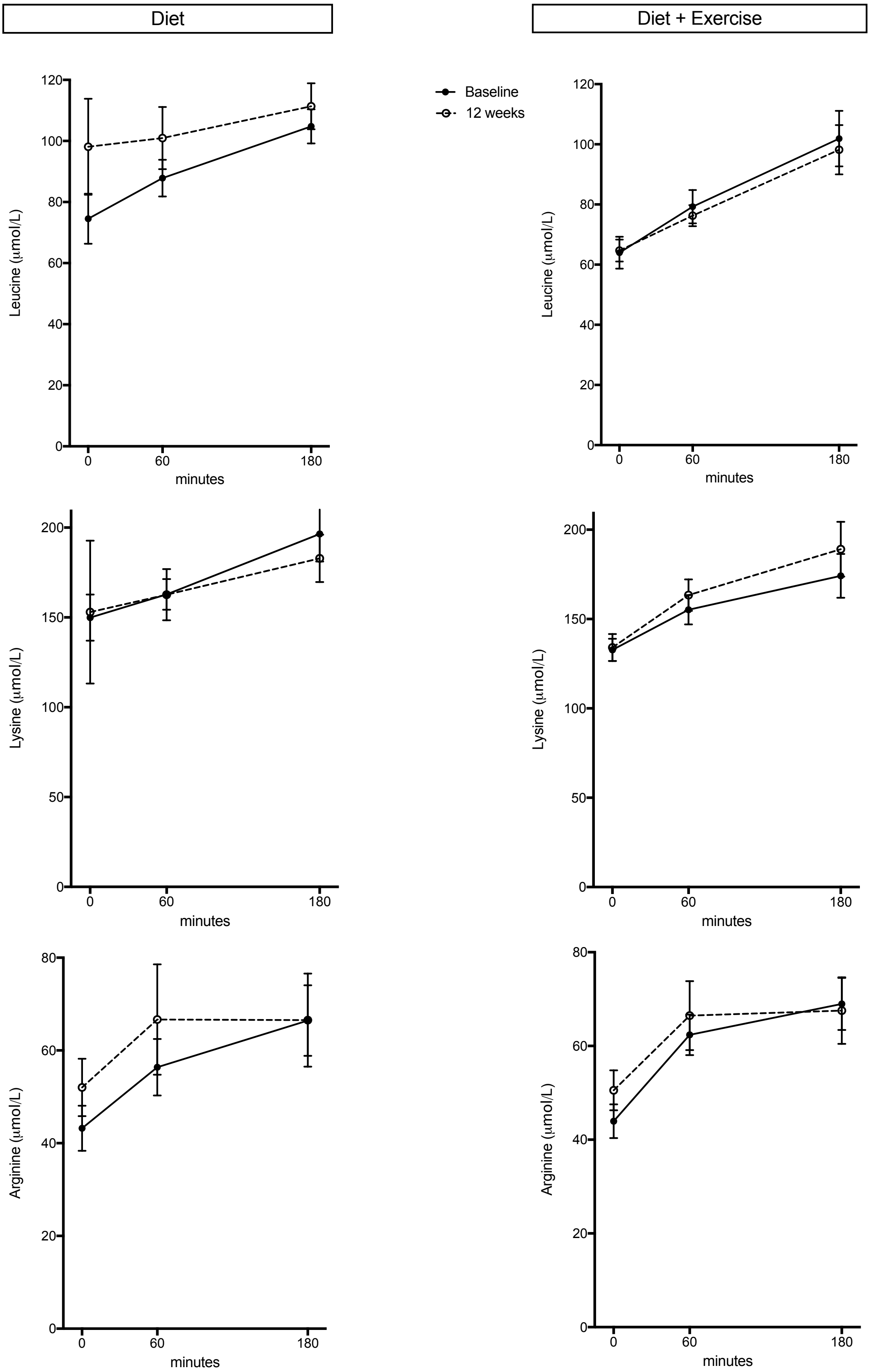
Diet
Diet + Exercise
Baseline
12 weeks
Leucine (μmol/L)
Lysine (μmol/L)
Arginine (μmol/L)
minutes
0
60
180
0
20
40
60
80
100
120
50
150
200

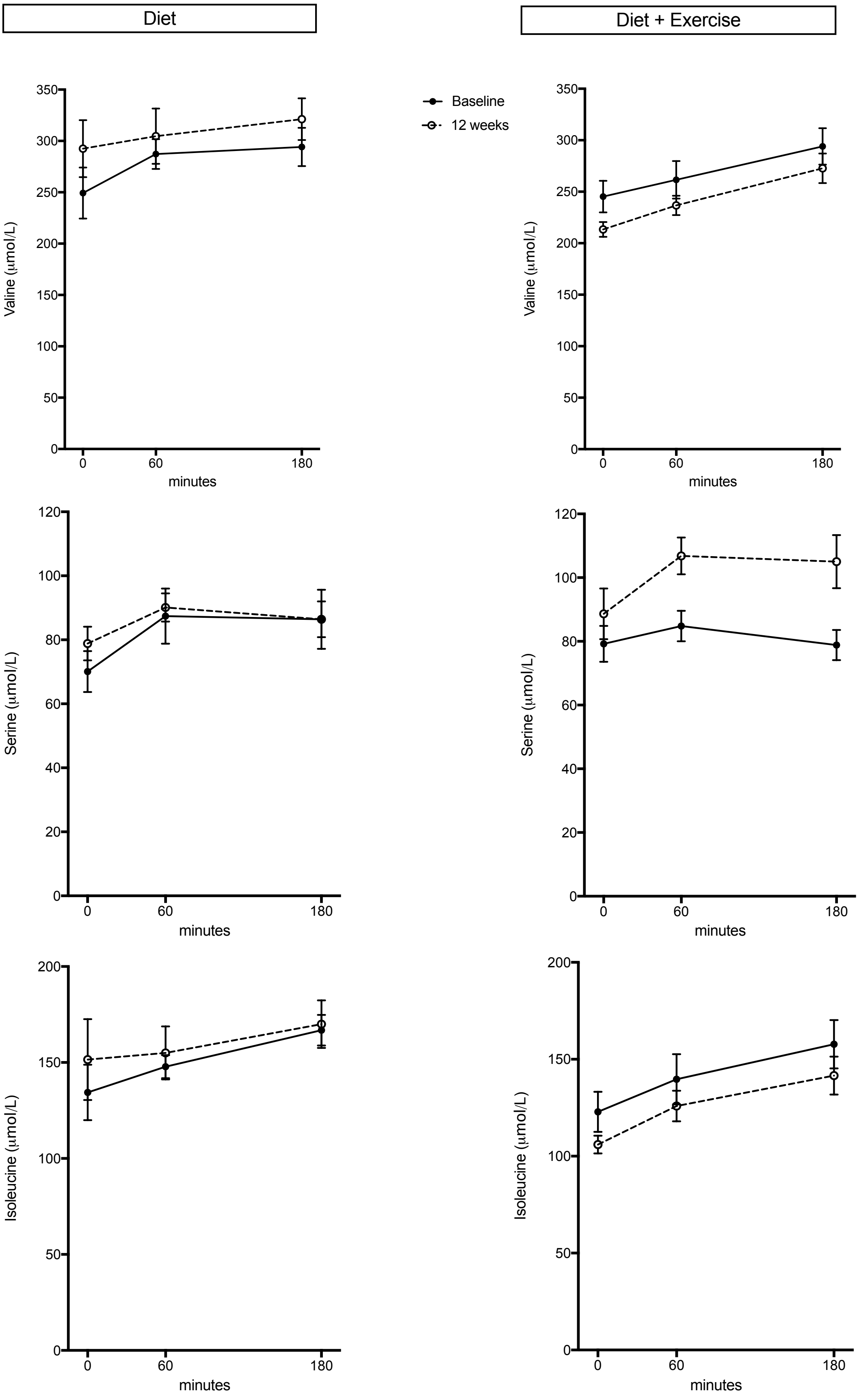
Diet
Diet + Exercise
Baseline
12 weeks
Valine (μmol/L)
Serine (μmol/L)
Isoleucine (μmol/L)
minutes
0
60
180
350
300
250
200
150
100
50
120
80
40
20

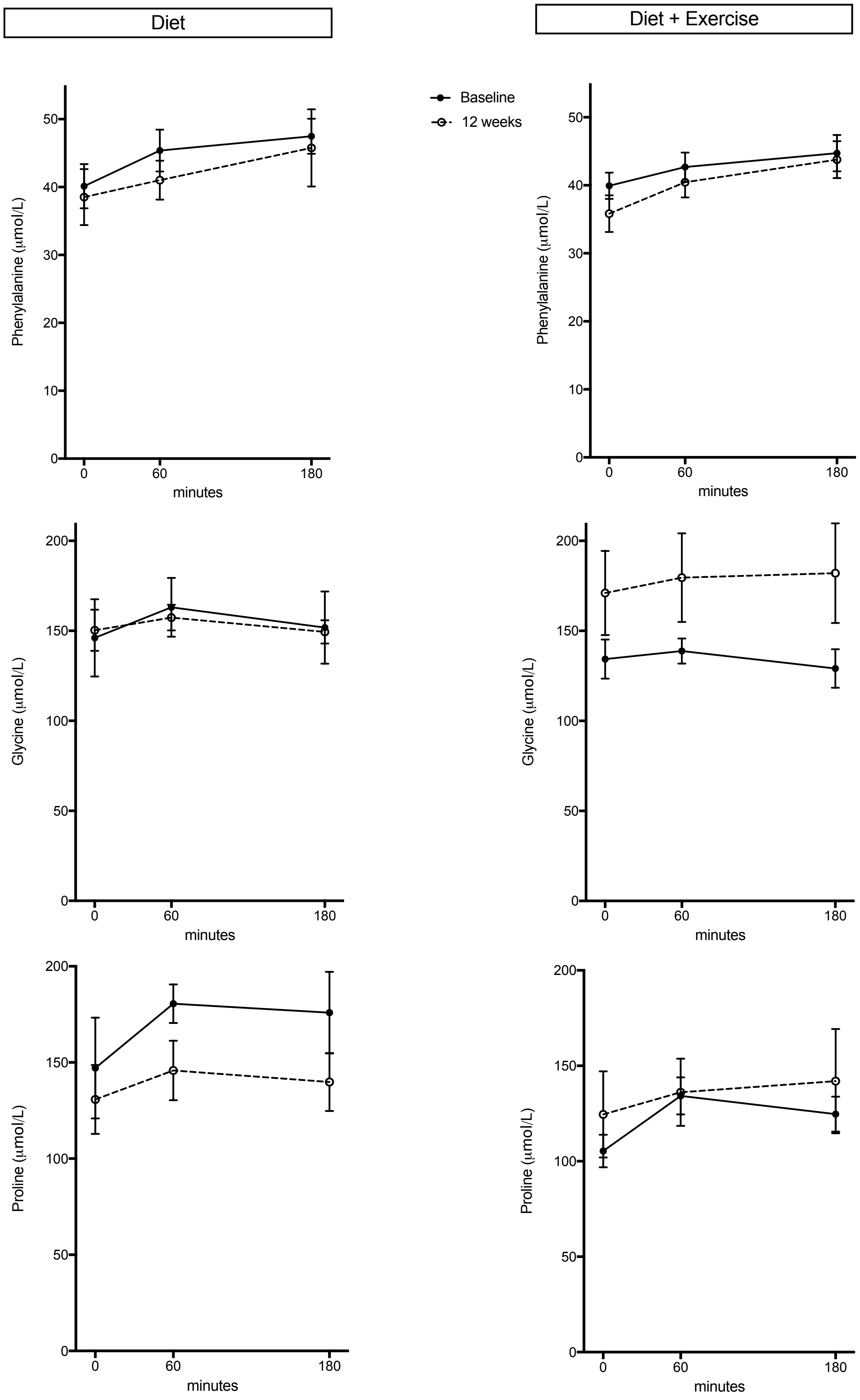
Diet
Diet + Exercise
Baseline
12 weeks
Phenylalanine (μmol/L)
Glycine (μmol/L)
Proline (μmol/L)
minutes
0
60
180
0
10
20
30
40
50
100
150
200

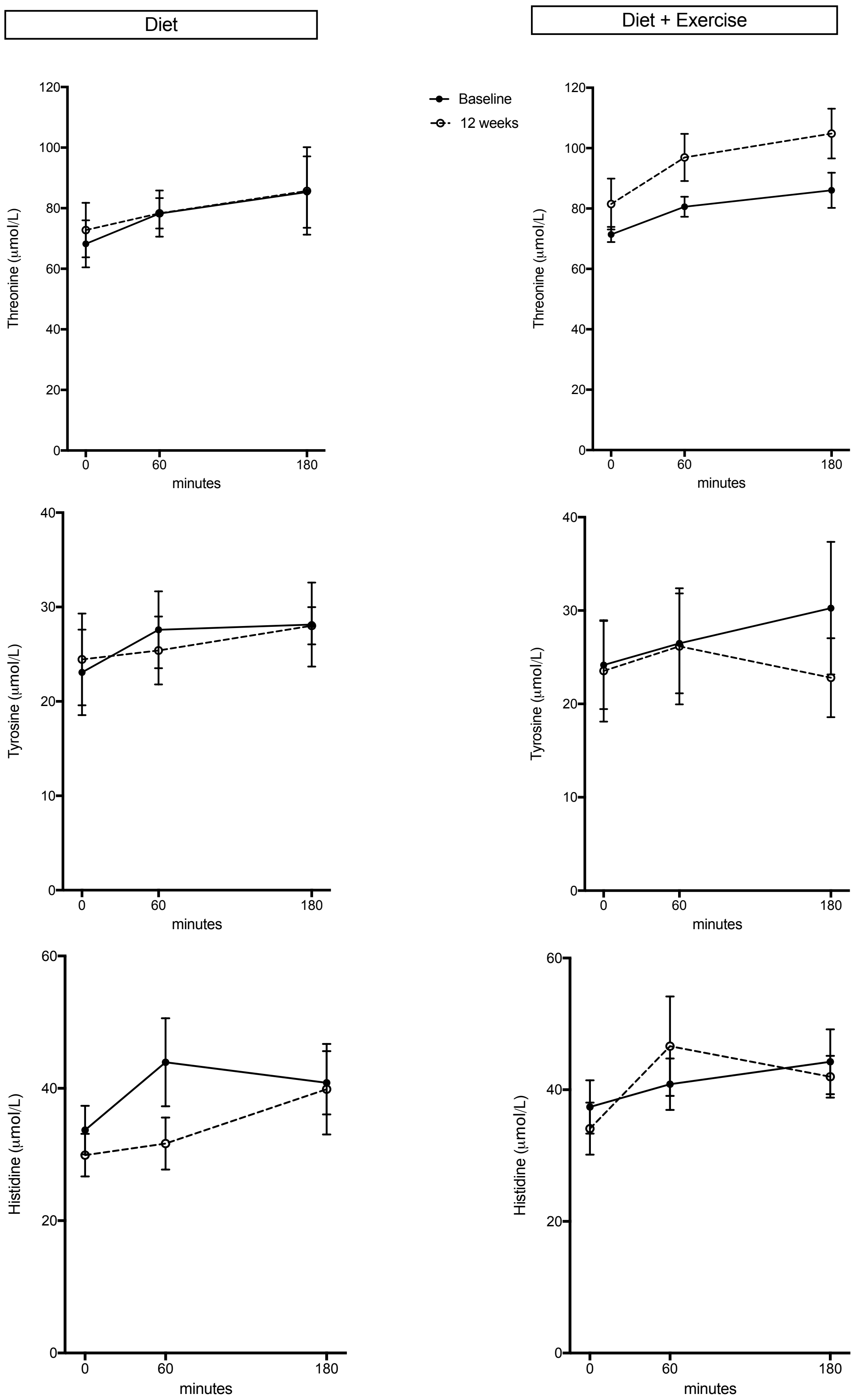
Diet
Diet + Exercise
Baseline
12 weeks
Threonine (μmol/L)
Tyrosine (μmol/L)
Histidine (μmol/L)
minutes
0
60
180
120
100
80
60
40
20
0
30
10

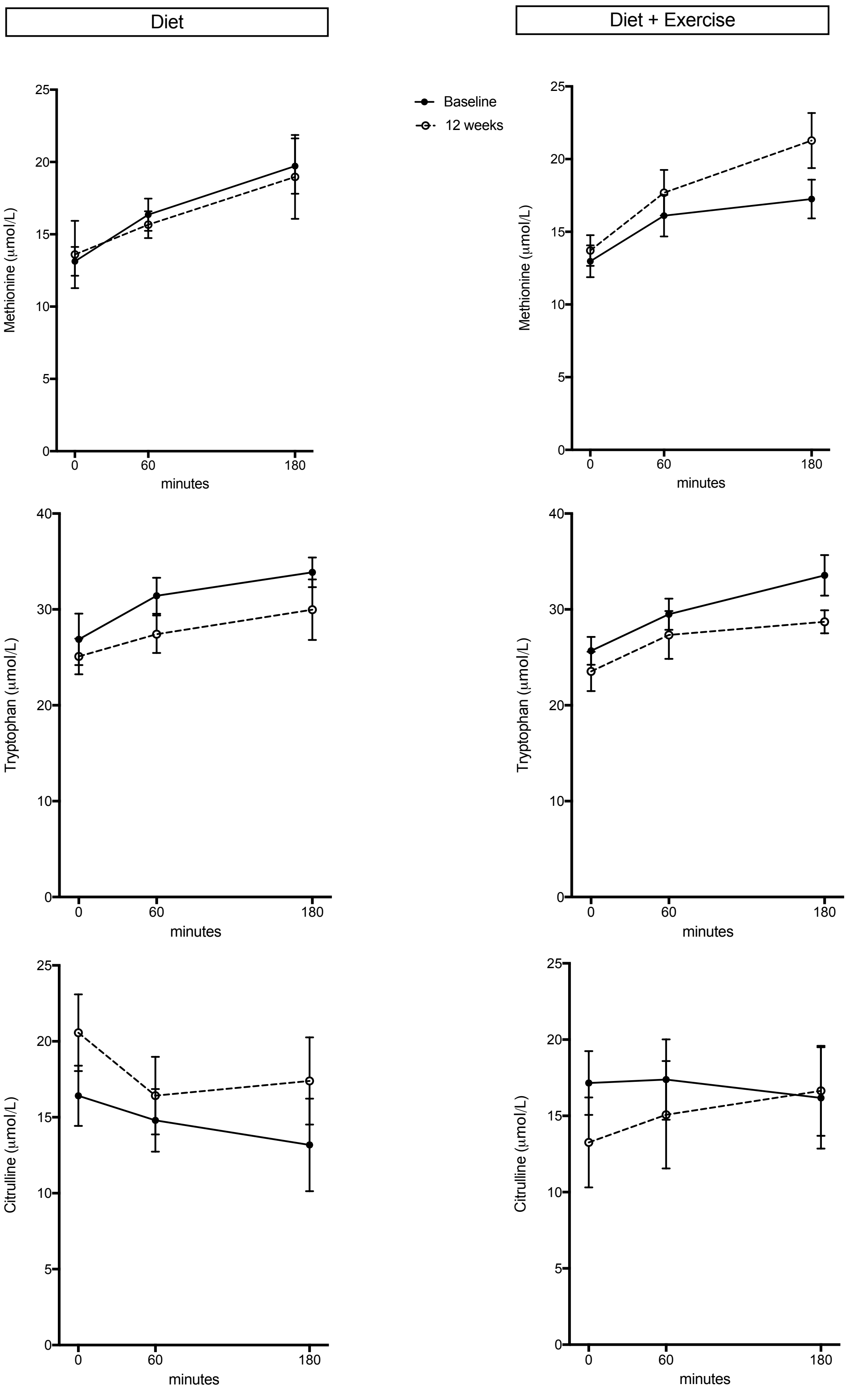

Diet
Diet + Exercise
Baseline
12 weeks
Methionine (μmol/L)
Tryptophan (μmol/L)
Citrulline (μmol/L)
minutes
0
60
180

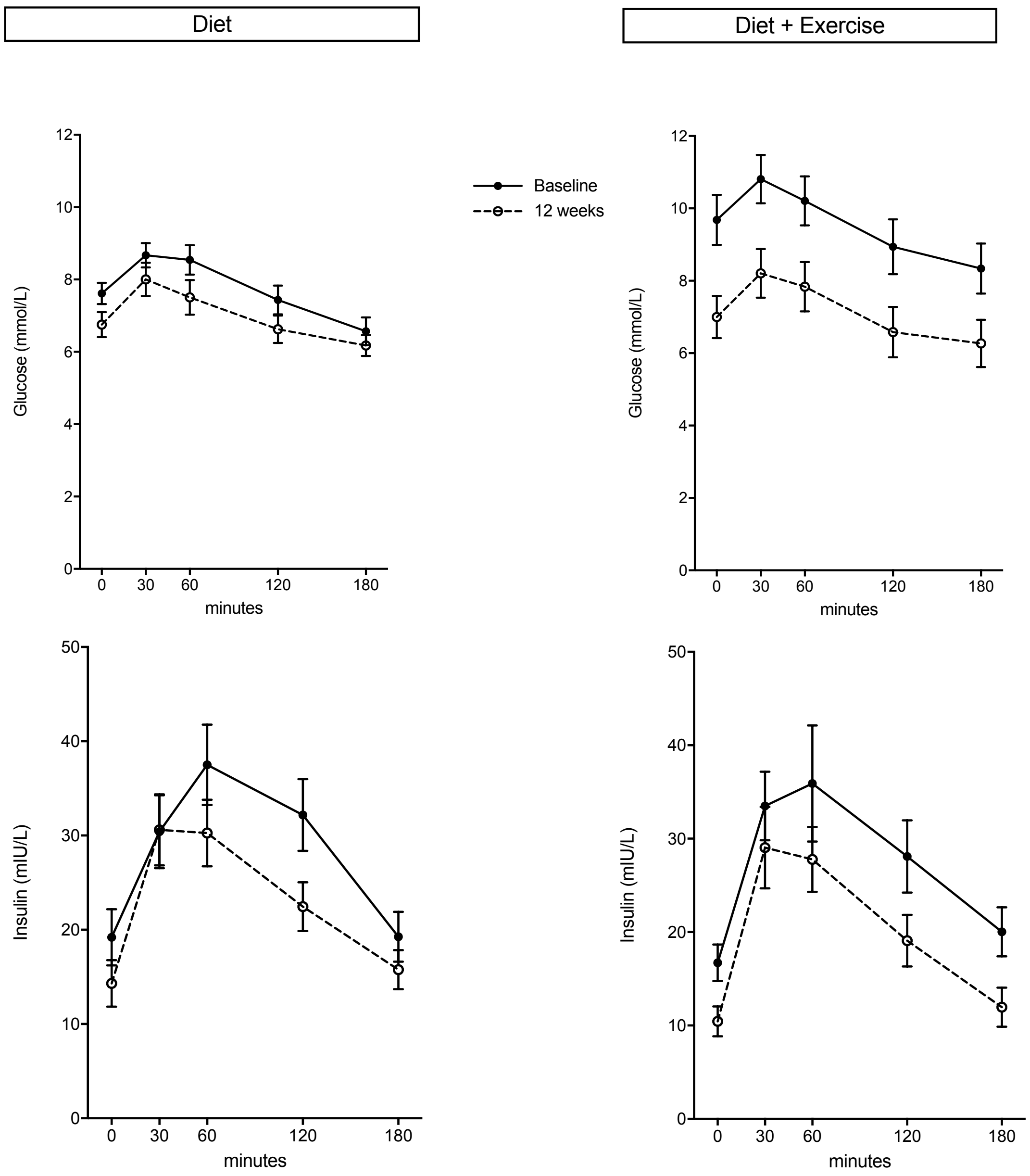

Diet
Diet + Exercise
Baseline
12 weeks
Glucose (mmol/L)
Insulin (mIU/L)
minutes
0
30
60
120
180
0
2
4
6
8
10
12
0
10
20
30
40
50
